# The effect of enamel proteins on erosion

**DOI:** 10.1038/srep15194

**Published:** 2015-10-15

**Authors:** T. Baumann, T. S. Carvalho, A. Lussi

**Affiliations:** 1 Department of Preventive, Restorative and Pediatric Dentistry, University of Bern, Freiburgstrasse 7, CH-3010, Bern, Switzerland

## Abstract

Enamel proteins form a scaffold for growing hydroxyapatite crystals during enamel formation. They are then almost completely degraded during enamel maturation, resulting in a protein content of only 1% (w/v) in mature enamel. Nevertheless, this small amount of remaining proteins has important effects on the mechanical and structural properties of enamel and on the electrostatic properties of its surface. To analyze how enamel proteins affect tooth erosion, human enamel specimens were deproteinated. Surface microhardness (SMH), surface reflection intensity (SRI) and calcium release of both deproteinated and control specimens were monitored while continuously eroding them. The deproteination itself already reduced the initial SMH and SRI of the enamel significantly (p < 0.001 and p < 0.01). During the course of erosion, the progression of all three evaluated parameters differed significantly between the two groups (p < 0.001 for each). The deproteinated enamel lost its SMH and SRI faster, and released more calcium than the control group, but these differences were only significant at later stages of erosion, where not only surface softening but surface loss can be observed. We conclude that enamel proteins have a significant effect on erosion, protecting the enamel and slowing down the progression of erosion when irreversible surface loss starts to occur.

Enamel is the hardest substance of the body, consisting of 96% (w/v) minerals, 3% water and 1% proteins^1^. The mineral portion is made up of carbonated hydroxyapatite, which forms long, thin crystallites, referred to as ribbons. These ribbons are approximately hexagonal in cross section and have a mean thickness of 26 nm and width of 68 nm^2^. The spaces between the ribbons are 1–2 nm wide and are filled with an organic matrix. Approximately 10000–40000 of such ribbons form a rod, sometimes also called prism^3^. These rods are arranged in a columnar fashion and they are also partly surrounded by a thin sheath of organic matrix called the rod sheath or sheath space^4^. So while making up only 1% of the weight of enamel, the organic matrix is very finely interspersed throughout the structure of enamel, both within and between the rods. The organic matrix is composed of proteins that were secreted by ameloblasts during enamel formation. They are thought to form a scaffold for the growing hydroxyapatite ribbons and are therefore necessary for proper enamel formation. These enamel proteins are later almost entirely reabsorbed by the same ameloblasts that secreted them. So, the remaining proteins could be regarded as “leftovers” and not a real part of the final mature product^5^. However, this is not the case, as they have important effects on the mechanical properties of enamel, influencing properties such as hardness and fracture toughness^6^.

Considering the effects they have on critical properties of enamel, it cannot be denied that enamel proteins are an important component of mature enamel. A few studies have investigated whether these proteins fulfill functional roles besides structural and mechanical ones. The effect of enamel deproteination on composite bond strengths for orthodontic bracket bonding has been studied. It has been suggested that enamel deproteination before acid etching increases the “conditioning” of the enamel surface^7^ and resin quality penetration^8^. Enamel deproteination could thus be leading to increased susceptibility to attack by acid agents. Another study showed that the average surface charge density of enamel is shifted in the positive direction by deproteination. In an acidic environment, this effect becomes even more pronounced. The result is a slightly positively charged surface instead of an increasingly negatively charged surface, which would normally be the case upon reduction of the pH of the tooth environment. The authors claim that this effect disrupts the “natural protection that occurs through the attraction of positive ions of calcium from saliva and delivering these ions to the enamel layer,” and that positively charged diffuse layers near the tooth surface will no longer be neutralized^9^. The same authors then showed that enamel proteins indeed mitigate the effects of an acid attack on enamel, reducing nanohardness loss, erosion depth and the loss of structural order^10^. Enamel proteins thus seem to play a significant role in protecting enamel in acidic environments.

Tooth erosion is defined as the dissolution of dental hard tissue due to chemical processes without the involvement of bacteria^11^. Consumption of acidic foods and beverages is the main extrinsic factor responsible for erosion. The increasing consumption of such foods and beverages has led to an increasing number of patients suffering from dental erosion^11^,^12^. When the outermost part of the teeth is affected during enamel erosion, the hydroxyapatite is dissolved by an acid. This leads to a release of calcium and phosphate and thereby to a demineralization and softening of the surface of the enamel^13^. The continued impact of acid on the enamel surface leads to bulk mineral loss and, eventually, surface loss. In addition, the softened surface layer is also more prone to abrasion (e.g. by tooth brushing^14^,^15^,^16^ or by friction from the tongue^17^), leading to irreversible surface loss of the affected teeth. Some protection against erosion is conferred by the salivary pellicle, which forms by specific adsorption of proteins, peptides and lipids from saliva onto enamel, but the exact level of this protection is difficult to assess^18^.

The aim of the present study was to further investigate the role enamel proteins play in protecting enamel from erosion. Because these proteins influence surface, structural and mechanical properties, it was hypothesized that they would also have additional effects on erosion. We applied methods used in dental research to directly measure the erosion of sound and of deproteinated enamel and compared the behavior of the two groups.

## Materials and Methods

### Chemicals

Lanthanum nitrate hexahydrate (La(NO_3_)_3_·6 H_2_O, Sigma-Aldrich ChemieGmbH, Steinheim, Germany, ≥99%) was used for the calcium analysis by atomic adsorption spectroscopy. Chloramine T trihydrate solution (Sigma-Aldrich, ≥98%) was used for the storage of extracted teeth as received. Citric acid (Merck, Stettlen, Switzerland, ≥99.5%) was used for the preparation of erosive solutions. Hydrazine hydrate (Sigma-Aldrich, Cat. No. 225819) was used to deproteinate the enamel samples.

### Sample preparation

Thirty-six human molars with caries-free buccal or lingual surfaces were selected from a pool of extracted teeth. All teeth were extracted by dental practitioners in Switzerland (no water fluoridation, 250 ppm F^−^ in table salt) and were stored in 1% (w/v) chloramine T trihydrate solution. Before the extraction, the patients were informed about the possibility of using their teeth for research purposes, and consent was obtained. Because we are using teeth from a pooled bio-bank, the local ethics committee categorized the samples as “irreversibly anonymised”, and no previous approval was necessary. The present experiment was carried out in accordance with the approved guidelines and regulations of the local ethical committee (Kantonale Ethikkommission: KEK). “The crowns of all teeth were separated from the roots using an Isomet Low Speed Saw (Buehler, Duesseldorf, Germany) and randomly divided into two groups. To create planar parallel surfaces, these crowns were embedded in resin (Paladur; Heraeus Kulzer, Hanau, Germany) in two planar parallel molds. The enamel surface was placed in the thinner mold (100 μm thick), over which we placed the thicker mold (5 mm), and embedded the specimen with the resin. The thinner mold was then removed, creating an overlay of 100 μm that was serially abraded under constant tap-water cooling using a Knuth Rotor machine (LabPol 21; Struers, Copenhagen, Denmark) with silicon carbide paper discs of grain size 18, 8, and 5 μm. This procedure eliminated the top 100 μm of the enamel surface layer, while the teeth were still maintained in the thicker mold (5 mm thick). Then, the molds were turned around and the opposite sides were ground under constant tap-water cooling with silicon carbide paper discs of grain size 46, 30 and 18 μm until reaching the mold. The embedded crowns were then taken out of the molds and the enamel surfaces were polished for 60 s with 3 μm diamond abrasive and another 60 s with 1 μm diamond abrasive on a Struers polishing cloth under constant cooling (LaboPol-6, DPMol Polishing, DP-Stick HQ; Struers). Between all polishing steps and after the final polish, all slabs were ultrasonicated for 1 min in tap water and rinsed. After the final polishing step, the specimens in the experimental group (n = 18) were removed from the resin, as the latter would interfere with the deproteination procedure. Because the embedding will not have any influences on the properties of the enamel surfaces that were tested in this study, the control group (n = 18) was left embedded in resin for ease of sample preparation and handling. The grinding and polishing procedure resulted in tooth specimens with a flat ground enamel area with a 100 μm cut-off layer on one side and a planar parallel flat ground surface on the other side.

### Deproteination

To remove proteins and peptides from the enamel specimens in the experimental group, the specimens were immersed in hydrazine hydrate^9^. Each specimen was placed individually in a 15 ml polypropylene tube, 2 ml of hydrazine hydrate was added and the specimens were incubated at room temperature (RT) for 1 h. After 1 h, the hydrazine hydrate was replaced with a fresh 2 ml aliquot and the specimens were placed in a heating cabinet at 70 °C. The hydrazine hydrate was changed again after a 1 h, 8 h and 23 h incubation at 70 °C. From then on, the hydrazine hydrate was changed every 24 h for another 13 days, maintaining the temperature at 70 °C, resulting in a total of 14 days of incubation in hydrazine hydrate at 70 °C. To remove all residual hydrazine, the specimens were washed successively in 50%, 75% and 96% ethanol at RT for 30 min each, respectively, and then they were rehydrated in mineral solution for several hours (1.5 mmol/l CaCl_2_, 1.0 mmol/l KH_2_PO_4_, 50 mmol/l NaCl, pH 7.0^19^). To cover the bottom surface, the deproteinated specimens were glued to 5 mm thick resin disks using super glue. The entire tooth specimens, except for the ground and polished enamel area, were coated with nail varnish. The finished samples therefore presented a defined exposed enamel area and, as a result of the grinding, planar parallel surfaces.

To exclude any effect of the elevated temperature on the enamel specimens, the control specimens were also stored at 70 °C for 14 days, in mineral solution. The mineral solution has no effect on the enamel crystals, but mineral deposits may form on the surface. These deposits are only loosely attached and can easily be removed by polishing. Therefore, the specimens were polished again with 1 μm diamond abrasive after the storage period to remove any deposits that might have formed.

### Calculation of the exposed enamel surface area

From every specimen, an image of the exposed enamel was taken under a light microscope (Leica M420; 12.5 × magnification). The open enamel area was manually outlined (circled) using the software program IM500, which further calculated the exposed surface area, taking into account the corresponding magnification factor of the microscope.

### Erosion

The prepared specimens were immersed in 10 ml of citric acid (1% (w/v), pH 3.6) for a total of 6 min under constant agitation (70 U/min, Salvis, Reussbühl, Switzerland) at 25 °C. The citric acid was changed at 1, 2, 4 and 6 min. At these times, the samples were removed from the acidic solutions, rinsed with deionized water (20 s) and dried with oil-free air (5 s). The acidic solutions were stored for calcium analysis at a later time, while the surface microhardness and specular reflection of the samples were measured immediately. After these measurements, erosion was continued in fresh citric acid until reaching the next time point.

### Surface microhardness (SMH) measurement

A Knoop diamond under a load of 50 g and a dwell time of 10 s was used to perform six different indentations at 25 μm intervals (MHT-10 Microhardness Tester; Anton Paar, Graz, Austria). The lengths of the long axes of the indentations were measured using an optical analysis system (Leica DMR Microscope; Leica Mikroskopie und Systeme, Wetzlar, Germany) and used to calculate the SMH (the average value from the six indentations). This procedure was performed before the first erosion (initial SMH) and after each erosive cycle. Relative SMH (rSMH) at time t was calculated using the formula rSMH = (SMH_t_/SMH_i_ * 100), where SMH_i_ is the initial SMH before any erosive challenge and SMH_t_ is the value at time t.

The lengths (L) of the long axes of the indentations were further used to calculate the depths (D) of the indentations according to the formula *D* = *L*/2 * tan *α*, where *α* = 3.75 deg, a known parameter of the Knoop diamond indenter.

### Analysis of calcium loss

The acidic solutions used during enamel erosion were analyzed to determine the amount of calcium ions released from the enamel surfaces. Analysis was carried out by means of a flame atomic absorption spectrometer (AAnalyst 400, Perkin Elmer Analytical Instruments, Waltham, MA, USA) equipped with acetylene-air gas input. Lanthan nitrate (5% (w/v), aqueous solution) was used in the atomic adsorption analysis of calcium to eliminate the interference of phosphate ions on the detection signal. The measured calcium concentrations were normalized to the corresponding enamel surface areas.

### Surface reflection intensity (SRI) measurement

The assembly of the device and the measurement procedure of the specular surface reflection intensity were described in previous publications^20^,^21^. Briefly, the samples were placed on a sample holder in such a way that the beam of light from the light source was localized in the middle of the exposed enamel surface. The specular reflection of the enamel surfaces was then recorded at a fixed angle of 45 degrees to the surface, at a wavelength of 635 nm. The position of the sample holder was adjusted for every sample to obtain the maximal surface reflection intensity. This point represents the best positioning of the sample in relation to the reflectometer, and it is expressed as a SRI value (enamel reflection factor, erf). In practical terms, higher SRI values represent a greater reflection intensity of the enamel surface. The reflection intensities of each sample were recorded initially before the first erosion and after each erosive cycle. Relative SRI (rSRI) at time t was calculated using the formula rSRI = (SRI_t_/SRI_i_ * 100), where SRI_i_ is the initial SRI before any erosive challenge and SRI_t_ is the value at time t.

### Statistical data analysis

All statistical results were calculated with the software program R 3.1.0. Shapiro-tests were performed to determine the normality for all data sets. As none of them showed a normal distribution of the values, analysis of progression differences was performed by non-parametric ANOVA for longitudinal data. Wilcoxon rank sum tests were performed for the comparison of the values between the groups at the different time points. Significance levels were set at p < 0.05.

## Results

All the exact p-values calculated for the differences between the deproteinated and control groups, for the absolute as well as for the relative values, at each time point, are summarized in Table 1.

**Figure 1:**
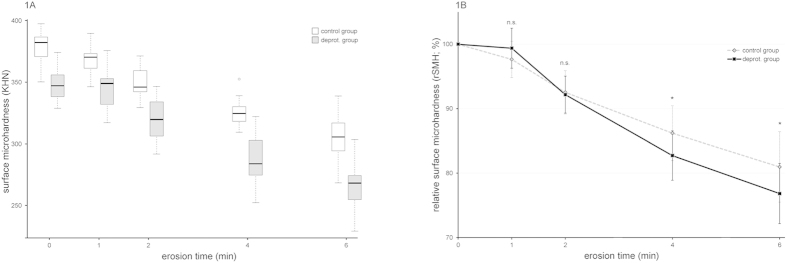
Microhardness of deproteinated and control enamel during the course of erosion. **(A)** Box plot of absolute Knoop surface microhardness values: the deproteinated enamel exhibited a significantly lower surface microhardness at all time points (p < 0.001). **(B)** Relative Knoop surface hardness values (±SD): the progression of the surface softening differed significantly between the two groups (p < 0.001), with the deproteinated enamel showing a faster softening of the surface. Wilcoxon rank sum tests for relative microhardness: *p < 0.05; n.s., not significant. Exact p-values can be found in Table 1.

Initial Knoop microhardness measurements revealed that the deproteinated enamel samples had significantly lower SMH values than the control group (Fig. 1A, p < 0.001). Because the two groups showed a significant difference in absolute values initially, we analyzed the relative microhardness values as well as the progression of the SMH during erosion. The analysis of the relative values revealed significant differences between the deproteinated and the control group after 4 and 6 min of erosion (p < 0.05), while also the overall progression of the values differed significantly (Fig. 1B, p < 0.001). The deproteinated enamel, therefore, showed a faster decrease in SMH during the course of the experiment, indicating faster surface softening and faster onset of the erosion process. The depths of the indentations created ranged from 1.37–1.48 μm initially to 1.49–1.73 μm after 6 min of erosion in the control group and from 1.41–1.55 μm initially to 1.57–1.92 μm after 6 min of erosion in the deproteinated enamel.

**Figure 2:**
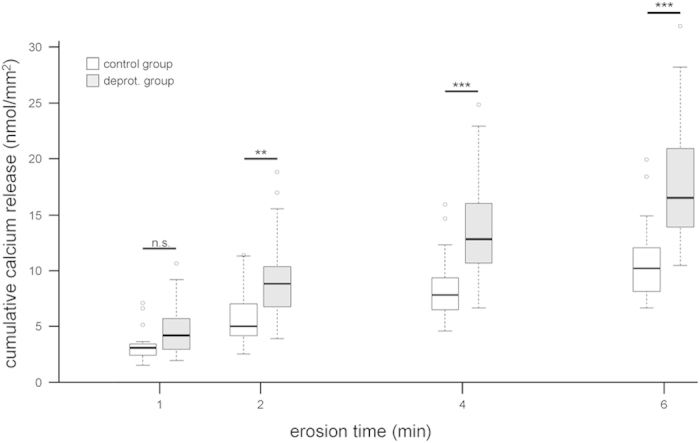
Calcium release of deproteinated and control enamel during the course of erosion. Deproteinated enamel started to release significantly more calcium upon erosion after 2 min and continued to release more thereafter, resulting in a significantly different progression of the calcium release between the two groups (p < 0.001). Wilcoxon rank sum tests: **p < 0.01; ***p < 0.001; n.s., not significant. Exact p-values can be found in Table 1.

Atomic absorption spectroscopy analysis of the calcium released from the enamel samples during each erosive cycle showed that the deproteinated enamel released significantly more calcium during the erosive challenges than the control group (Fig. 2). While at 1 min the difference was not significant, it became significant at 2 min of erosion (p < 0.01). At later times, the differences became even more obvious (p < 0.001 after 4 and 6 min). These differences also resulted in a significantly different progression of calcium release, with the deproteinated enamel releasing calcium at a higher rate than the control group (p < 0.001).

**Figure 3:**
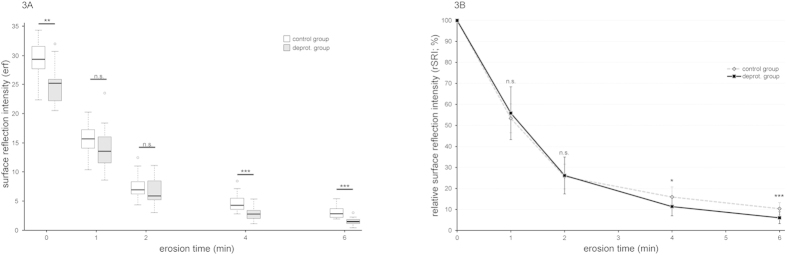
Surface reflectivity intensities (SRIs) of deproteinated and control enamel during the course of erosion. **(A)** Box plot of absolute intensity values. The deproteinated enamel exhibited a significantly lower SRI initially and after 4 and 6 min, but not at 1 and 2 min of erosion. **(B)** Relative intensity values (±SD). The progression of the SRI differed significantly between the two groups (p < 0.001) due to the differences at later stages of erosion, with the deproteinated enamel showing a faster decrease. Wilcoxon rank sum tests: *p < 0.05; **p < 0.01; ***p < 0.001; n.s., not significant. Exact p-values can be found in Table 1.

Similar to SMH, the initial SRI of the two groups also differed significantly (p < 0.01). Figure 3A shows that after the difference in the initial values, the values at 1 and 2 min of erosion were not significantly different between the groups anymore, while at 4 and 6 min they became significantly different again (p < 0.001). However, because the two groups showed a significant difference in absolute values initially, we analyzed the relative SRI values and the progression of the values (Fig. 3B). A comparison of the two groups showed that the deproteinated group lost more of its SRI during the course of erosion, resulting in a significantly different progression in the SRI loss of the two groups (p < 0.001). Similar to the relative SMH values, the differences in relative SMI between the groups were not significant in the first 2 min of erosion but became significant thereafter (p < 0.05 after 4 min, p < 0.001 after 6 min).

## Discussion

The deproteination protocol used here is a modification of the original protocol proposed by Termine and coworkers to deproteinate bones^22^. We chose this protocol because it is regarded as one of the most effective deproteination methods and it does not alter the mineral morphology or composition^23^. It has been validated for enamel, showing that this method removes proteins from the surface layers of enamel almost completely, while leaving the carbonate content unaltered and preserving the ordered arrangement of hydroxyapatite crystallites within the enamel rods, indicating no structural changes^9^,^10^. The deproteination of the specimens, therefore, created an enamel surface structure containing empty spaces between the single crystallites and between prisms and the interprismatic enamel, where the rod sheath used to be. Unfortunately, we were not able to measure the exact depth of protein removal in the present study. The maximal depth of the indentations created for the hardness measurements was 1.92 μm. This is within the range of previously reported depths of hardness measurements in deproteinated enamel using the same deproteination protocol^10^. The temperature and long incubation time of the protocol assures a deproteination within the outer surface layers investigated here and that should reach at least these depths.

The role of proteins in mature enamel has, so far, mainly been studied with regard to structural and mechanical properties of enamel. We wanted to study the effects enamel proteins have on dental erosion, especially on the onset of erosion. This initial erosion is characterized by changes on the tooth surface, such as surface softening, before a loss of tissue is observed^24^. When exactly tissue loss can be observed, and which method is best suited to measure it, is still a matter of debate. In the present study, we chose an erosion time of no more than 6 min, as we know that this already results in slight surface loss. Furthermore, the effects of erosion detectable by the methods used here tend to level off with increasing substance and surface loss^20^. Therefore, longer erosion times would have diminished any differences between the groups and would not have allowed for the detection of early erosion effects. In addition, severe substance loss caused by longer erosion times might have exposed deeper regions in the enamel where the deproteination would not have been assured anymore.

An extensive comparison of the buccal and lingual surfaces of the same teeth has revealed that there are no significant differences detectable between these two sides with the methods used in the present study^25^. Therefore, we made no distinction between buccal and lingual sides when preparing the specimens for the experiment.

To measure SMH, flat and highly polished surfaces are necessary. This makes it impossible to investigate the native surface of enamel using this method. The structure and composition of enamel, and therefore also its properties, will change as one moves from the tooth surface to the dentin–enamel junction^26^. Having that in mind, we removed as little enamel as possible from the enamel surface in order to investigate an enamel layer as closely related to the outer enamel as possible. For that, we standardized the depth of the enamel by grinding away exactly 100 μm of enamel. This created enamel surfaces big enough to measure SMH, SRI and calcium released. At the same time, with only such a thin layer of enamel having been removed, the enamel can still be considered as being nearly identical, in structure and composition, to the outermost prismatic enamel. The drawback of this kind of sample preparation is that the aprismatic or prismless enamel layer covering sound teeth is also removed. This aprismatic layer is more resistant to acidic attacks^27^, but it cannot be standardized for experimental purposes and shows natural variations between different individuals and different tooth sites.

The SMH results show that the deproteination of the enamel already softens the surface, resulting in significantly different initial hardness values (Fig. 1A). This seems to contradict other studies that have shown that deproteination increases the hardness of enamel^10^, and that hypomineralized enamel with a higher protein content is softer than normal enamel^28^. However, these studies used nanoindentations, which measure the hardness of single prisms. The crystallites within prisms are tightly packed and surrounded by a very thin protein layer which can act both as “glue” and as a lubricant. If this layer is removed, the friction between crystallites will increase and the loss of the lubricating effect will dominate the loss of the gluing effect, leading to higher hardness values of the prisms. In this study, we used microhardness measurements with a Knoop indentor, which creates indentations covering a larger area that spans several prisms, interprismatic regions and rod sheaths. The interprismatic enamel and rod sheaths contain a large amount of the enamel proteins, and it has been shown that these proteins form bridging elements between prisms^29^. They “glue” the crystallites and prisms together, dissipating some of the energy by deforming and unfolding when indentations are created, thus influencing hardness measurements^6^,^30^. As the crystallites in the interprismatic enamel are not as tightly packed as in the prisms and the rod sheath is much thicker than the protein layer surrounding crystallites, deproteination will create much larger gaps there than within the prisms. Because of these large gaps, the friction increase between prisms will be much less than between crystallites, and the loss of the gluing effect will dominate, allowing the prisms to slide along each other more easily, leading to lower overall hardness values of enamel.

Even though we observed different initial hardness values, the progression of the microhardness loss differed statistically significantly between the two groups during erosion. Figure 1B shows that the deproteinated group lost little of its original hardness in the first minute, but then softened at a faster pace than the control group. Over very short erosion times, only the softening of the top surface of the enamel prisms and crystallites appeared to contribute to the microhardness loss. This initial surface softening might be small in comparison to the effect that the deproteination has on the hardness. Therefore, the overall hardness might seem to remain rather constant in the beginning. However, at longer erosion times, the acid is able to penetrate deeper into the enamel, exploiting the gaps created by deproteination. This is different on the samples from the control group, which still have their organic sheaths preserved. While these organic sheaths surrounding the prisms are softer than the enamel prisms themselves, they are more resistant to acidic attacks^4^, denying the acid access to the sides of the dissolving prisms and interprismatic enamel. In deproteinated enamel, this barrier is missing and the acid can dissolve the minerals not only from the top of the crystallites and prisms, but also along the sides, explaining the faster rate of surface softening.

The analysis of the progression of calcium released from the two groups revealed that they behaved significantly differently (Fig. 2). The behavior generally showed the same pattern as the progression of the SMH. Although no significant differences were observed after 1 min, the differences appeared after 2 min of erosion and became highly significant thereafter. This fits the explanation given above for the softening of the surface. The additional calcium released most likely originated from the increased surface area in the deproteinated group. In this group, the absence of proteins exposed more of the crystals, and the acid could then attack and dissolve along the uncovered sides of the crystallites and prisms of deproteinated enamel, explaining the higher levels of released calcium.

Similar to microhardness, there was also a significant difference between the groups in the initial SRI values. Since the SRI is dependent on the roughness of a surface^30^, this difference may be explained by the removal of the proteins. The deproteination creates small gaps between crystallites and larger gaps between prisms, which render the surface rougher than in untreated control enamel, and thereby lower the SRI of the samples. When the samples were placed in acid, the initial erosion reduced this difference, as it led to an overall roughening of the surface. This effect was much bigger than the roughening created by the deproteination, which left the biggest part of the surface unaltered. During early erosion, the effect of the deproteination itself on the SRI values therefore became marginal, leading to non-significant SRI differences between the groups at 1 and 2 min of erosion. At later points of the erosion, the differences became significant as the dissolution rate of the enamel differed between the groups. To better visualize the differences in the progression of SRI loss, we plotted the relative SRI loss over time (Fig. 3B). The graph reveals almost identical patterns for the two groups in the first 2 min of erosion, but then the SRI of the deproteinated group dropped faster than that of the control group, resulting in a significantly different progression of the SRI loss of the two groups and indicating a faster rate of erosion in the deproteinated group.

Previous studies have shown that there are different phases of enamel erosion *in vitro*. Shellis and coworkers^31^ showed that erosion is a near-surface phenomenon. The acid is usually found in an equilibrium state, where both dissociated and undissociated acid molecule forms are present in the solution. The H^+^ ions first attack the enamel surface, kick-starting the erosion process and forming small pores on the enamel surface. These pores give access to the undissociated form of the acid to penetrate the enamel tissue, which then dissociates within the enamel itself, thus serving as a proton carrier^32^. This induces demineralization in the near-surface layer of the enamel, which leads, in turn, to surface softening^31^. In general terms, in the first 2 min, only surface softening takes place in the near-surface layer of enamel, while over longer erosion times more and more of the enamel is dissolved until surface loss also sets in^20^. The results of all three parameters observed in this study indicate that deproteination mainly affects the second phase of erosion, when the enamel starts dissolving to the extent that surface loss starts to occur. During the softening phase, no significant differences were observed in the progression of SMH and SRI values. We therefore hypothesize that the proteins in the enamel play a more substantial role in preventing enamel surface loss by slowing down the dissolution of the surface layers of enamel, rather than in inhibiting the initial near-surface demineralization and softening of the surface.

The protective effect could, on the one hand, be passive protection; simply limiting the access of acids to the top of the surface of enamel. The protein matrix is likely to be the main channel for ionic conduction in enamel^10^. By filling the small gaps between enamel crystallites and prisms, enamel proteins prevent acidic solutions from penetrating along the sides of these crystallites and prisms deep into enamel. This restricts the possible contact sites where the acid can dissolve the enamel to the very top surface of the prisms, on the enamel surface. On the other hand, enamel proteins might also protect the enamel in a more active manner. A study investigating the surface properties of enamel showed that untreated enamel possesses a net negative surface charge, which becomes increasingly negative in an acidic environment. The authors claim that this effect leads to natural protection by attracting calcium ions from saliva. When the enamel is deproteinated, this behavior is reversed. Compared to the untreated enamel, the surface charge of deproteinated enamel is already reduced in a neutral environment. In an acidic environment the negative charge is reduced even more and even switches to a positive surface charge^9^. Another way for enamel proteins to more actively protect the enamel from erosion might be by interacting with proteins from saliva, influencing the formation and composition of the pellicle. The pellicle forms almost instantaneously upon contact of saliva with enamel surfaces. Initially, pellicle proteins adsorb onto enamel mainly because of electrostatic interactions with calcium and phosphate ions of the hydroxyapatite surface^18^, while van der Waals forces and hydrophobic interactions also contribute to the adsorption^33^. Besides hydroxyapatite, also the enamel proteins might play a role in these processes, which could lead to a stronger (or weaker) adsorption of pellicle proteins or a change in the composition of the pellicle. As we have shown in the present study, protection by enamel proteins had its main effect at later stages of erosion, where substance loss can be observed and even surface loss starts to set in. Active protection because of a change of electrostatic properties of the surface should not only work at a later stage of erosion, but also in the beginning. In that case, a passive form of protection seems more likely. Still, an active form of protection cannot be excluded since our model did not include saliva or calcium ions, which would be necessary for the proposed active protections.

## Conclusions

The enamel proteins present in mature enamel do not only influence the mechanical properties of enamel, but also affect resistance to acid attacks. The natural presence of proteins in enamel in our experimental set-up likely leads to a passive protection that limits the access of the acid to the top of enamel crystallites, thereby decreasing the progression of erosion.

## Additional Information

**How to cite this article**: Baumann, T. *et al.* The effect of enamel proteins on erosion. *Sci. Rep.* **5**, 15194; doi: 10.1038/srep15194 (2015).

## Figures and Tables

**Table 1:** Summary of p-values for differences between groups of all investigated parameters.

	initial	1 min	2 min	4 min	6 min
absolute SMH values	<0.0001	0.0001	<0.0001	<0.0001	<0.0001
relative SMH values	—	0.1211	0.8619	0.0220	0.0218
absolute Ca^2+^-release	—	0.0689	0.0071	0.0004	0.0003
absolute SRI values	0.0013	0.0818	0.1813	0.0003	0.0001
relative SRI values	—	0.5269	0.8173	0.0100	0.0007

Wilcoxon rank sum tests were performed to analyze differences of absolute and of relative values between the groups at all time points of erosion. Significance levels were set at p < 0.05.
